# Long-Term Risk of Death From Heart Disease Among Breast Cancer Patients

**DOI:** 10.3389/fcvm.2022.784409

**Published:** 2022-04-13

**Authors:** Aya Agha, Xi Wang, Ming Wang, Eric J. Lehrer, Samantha R. Horn, Jennifer C. Rosenberg, Daniel M. Trifiletti, Roberto Diaz, Alexander V. Louie, Nicholas G. Zaorsky

**Affiliations:** ^1^Department of Radiation Oncology, Penn State Cancer Institute, Hershey, PA, United States; ^2^Department of Public Health Sciences, Penn State College of Medicine, Hershey, PA, United States; ^3^Department of Radiation Oncology, Icahn School of Medicine at Mount Sinai, New York, NY, United States; ^4^Department of Radiation Oncology, Mayo Clinic, Jacksonville, FL, United States; ^5^Department of Radiation Oncology, H. Lee Moffitt Cancer Center and Research Institute, Tampa, FL, United States; ^6^Department of Radiation Oncology, Odette Cancer Centre – Sunnybrook Health Sciences Centre, Toronto, ON, Canada; ^7^Department of Radiation Oncology, University Hospitals Seidman Cancer Center, Case Western Reserve School of Medicine, Cleveland, OH, United States

**Keywords:** cardio-oncology, radiotherapy, breast cancer, heart disease, chemotherapy

## Abstract

**Background:**

Most breast cancer patients die of non-cancer causes. The risk of death from heart disease, a leading cause of death, is unknown. The aim of this study is to characterize the long-term risk of fatal heart disease in breast cancer patients.

**Methods:**

This retrospective study used the Surveillance, Epidemiology, and End Results (SEER) database. Standard mortality ratios (SMR) were calculated for breast cancer patients diagnosed from 1992 to 2014. Patients were stratified by receipt of radiotherapy and/or chemotherapy, disease laterality, and diagnosis era. Hazard ratios (HRs) and odds ratios (ORs) were calculated to compare the risk of death from heart disease among other breast cancer patients.

**Results:**

There were 1,059,048 patients diagnosed with breast cancer from 1992 to 2014, of which 47,872 (4.6%) died from heart disease. The SMR for death from heart disease at 10+ years for patients who received only radiotherapy was 2.92 (95% CI 2.81–3.04, *p* < 0.001) and in patients who received only chemotherapy was 5.05 (95% CI 4.57–5.55, *p* < 0.001). There was no statistically significant difference in SMR for death from heart disease for left-sided vs. right-sided disease. At 10+ years, heart disease made up 28% of deaths from non-primary cancer. HRs and ORs showed that the risk of death from heart disease was highest in patients older than 70 years of age and with longer follow-up.

**Conclusion:**

The risk of fatal heart disease was highest in older breast cancer patients with longer follow-up (i.e., >5–10 years) and who received chemotherapy. These patients should be referred to cardio-oncology clinics to mitigate this risk.

## Introduction

Breast cancer affects approximately 1 in 8 women in the United Sates, and is the second leading cause of cancer deaths in women (1). As more women survive breast cancer, there is an increased emphasis on the long-term consequences of radiotherapy and chemotherapy. Heart disease accounts for approximately 25% of deaths in the United States and is a serious complication of breast cancer treatment (2, 3). Stoltzfus et al. studied the overall risk of fatal heart disease among cancer patients and show that in almost all cancer types the risk increases with time (4). Patnaik et al. demonstrate the significant effect that comorbid conditions have on the long term outcome in breast cancer patients specifically, showing that with longer follow-up time, cardiovascular disease becomes the cumulative leading cause of death (5).

Both chemotherapy and radiotherapy are associated with increased risk of cardiac-related complications in cancer patients. Chemotherapeutic agents, in particular anthracyclines, have significant cardiotoxic effects. Doyle et al. show that patients receiving chemotherapy have a hazard ratio (HR) of 2.48 (95% CI 2.10–2.93) for cardiomyopathy (6). Boekel et al. found that breast cancer patients receiving chemotherapy after 1997 had an increased risk of developing congestive heart failure with a HR of 1.35 (95% CI 1.00–1.83) (7).

Radiation therapy also increases the risk of cardiovascular complications. Cheng et al. showed that radiation increased the risk of cardiac mortality in breast cancer patients with a relative risk of 1.38 (95% CI 1.18–1.62) compared to women who did not receive radiation (8). Furthermore, patients with left-sided breast cancer are thought to be at an increased risk of cardiovascular complications, compared to patients with right-sided disease. This is because left-sided disease and consequent radiation treatment to the internal mammary chain can increase the radiotherapy dose to the heart (9, 10).

This association between cancer therapy and cardiac complications has led to the development of the subspecialty of cardio-oncology, in order to better understand best practices for treating cancer patients while being cognizant of risks for cardiac disease. As breast cancer therapies improve and more patients survive breast cancer, these long-term complications become more significant and need to be considered fully when designing treatment plans.

The primary objective of this study is to characterize the risk of death from heart disease among breast cancer patients compared to the general United States population on the basis of treatment received, disease laterality and year of diagnosis. The secondary objective is to characterize the risk of death from heart disease within different subgroups of breast cancer patients. We define death from heart disease as any disease that occurs on the heart including myocardial infarction, heart failure, coronary artery disease, and arrhythmias. This study is unique from other retrospective studies due to the large sample size and duration of follow-up, further strengthening our analysis. This analysis is an important addition to the current body of literature as it helps inform the multi-disciplinary team on cardiac considerations during breast cancer survivorship. Additionally, this work highlights which patients are at the greatest risk of cardiac complications and can therefore help with personalizing treatment for patients with cardiac comorbidities. This work can also be helpful for creating guidelines regarding heart disease prevention among breast cancer patients for patients most at risk.

## Materials and Methods

This was a retrospective study using Surveillance, Epidemiology, and End Results (SEER) 13 and 18 databases. SEER collects data from 28% of the United States population *via* a network of population-based incident tumor registries from geographically distinct regions in the United States (11, 12). The SEER registry includes data on age at diagnosis, sex, race, marital status, and year of diagnosis. SEER*Stat 8.3.5 was used for analysis (11). Patients diagnosed with breast cancer between 1992 and 2014 were abstracted from the SEER program and stratified based on treatment received (radiotherapy only, chemotherapy only, both radiotherapy and chemotherapy, or neither radiotherapy nor chemotherapy), year of diagnosis and laterality of disease (11, 12). All patients with an invasive cancer diagnosis were included. Patients diagnosed with cancer *via* autopsy or death certificate (<1.5% of patients) were excluded. Time in SEER was measured in months, with the minimum time to any event equal to 1 month.

Surveillance, Epidemiology, and End Results assigns mortality codes for patients using death certificates that the treating physician completes. Cause of death is coded in SEER based off the International Classification of Diseases 9 (ICD-9) or ICD-10 which includes primary and secondary cancer sites, as well as non-cancer causes, for example heart disease. However, SEER does not further classify heart disease as a cause of death; therefore, the specific nature of the fatal heart disease is unknown. Additionally, important comorbidities are also unknown about the patients and these may act as confounding variables, examples being history of heart disease, cholesterol levels as well as smoking status. SEER codes receipt of radiotherapy *via* the modality used but does not give further specifics in terms of dosing. Similarly, receipt of chemotherapy is coded as yes or no/unknown and therefore does not provide information regarding which specific agents are used, limiting the extent of analysis that can be performed.

For the primary objective, standard mortality ratios (SMRs) with 95% confidence intervals, which provide relative risk of death from heart disease in a population under investigation vs. the general United States population (matched for age and race), were calculated for patients in the analysis. Our study examined patients from 1992 to 2014 in order to assess if the modern era radiation modality has impacted the rate of fatal heart disease among breast cancer patients. SMRs from different populations should not be compared to one another as they are dependent on the United States population during the time of interest, and therefore, if United States populations vary during those times it can interfere with the interpretation.

For the secondary objective, death from heart disease was considered as a binary variable. Odds ratios (ORs) and 95% CIs were calculated from the multivariate logistic regression models. Time to heart-disease-related death was calculated as time from diagnosis to death or last contact date, whichever happened first. With living patients being censored, we performed multivariable Fine and Gray’s subdistribution hazards model to account for competing risk of death from causes other than heart disease with HRs and 95% CIs calculated. We adjust for the same covariates in the multivariable logistic regression model and multivariable Fine and Gray’s subdistribution hazards model, including year of diagnosis, T stage, N stage, age, race, laterality, chemotherapy, and radiation therapy. Patients with missing values in the covariates were excluded from the logistic regression analysis giving *N* = 976,359. To perform the Fine and Gray’s model, we further excluded observations with missing survival time to get *N* = 975,988. Furthermore, in order to predict the risk of death from heart disease in breast cancer patients, a nomogram was developed based on the multivariate model for time-to-event outcomes (13).

The source data supporting the findings of this study are provided in the Supplementary Material. Any additional information may be requested from the corresponding author. The data obtained for the current project from the SEER database are freely accessible to the public. The relevant session information, i.e., the user-submitted request, from in the current work and abbreviated data set (from SEER) are provided in Supplementary Notes.

We comply with all relevant ethical regulations. The datasets generated and analyzed during the current study are available in the SEER repository.^1^ The study was exempt from IRB review as these data are freely available *via* the National Cancer Institute SEER program. There are no participants in the study, and thus there is no consent form.

## Results

There were 1,059,048 patients diagnosed with breast cancer from 1992 to 2014 of which 47,872 patients (4.6%) died from heart disease. Table 1 shows demographics for patients included in this analysis. There were 330,719 (31.2%) patients greater than 70 years old included in the study. The breakdown of patients with T1, T2, T3, and T4 disease was 563,596 (53.2%), 264,690 (25.0%), 44,764 (4.2%), and 30,755 (2.9%), respectively. Of the patients in this analysis, 411,272 (39%) had nodal involvement and 84,780 (8.2%) had metastatic disease. The primary disease site was right-sided in 531,536 patients (50.2%) and left-sided in 514,685 patients (48.6%). 449,318 (42.4%) patients received external beam radiation and 391,296 (36.9%) patients were treated with chemotherapy.

**TABLE 1:** Demographics for patients diagnosed with breast cancer from 1992 to 2014 and for patients who died from heart disease.

	Total diagnosis* (percentage)	Heart disease^†^ (percentage)
Total patients	1,059,048	47,872
**Age group**		
≤29	5,563 (0.5)	9 (0.02)
30–39	49,499 (4.7)	157(0.3)
40–49	177,711 (16.8)	929 (1.9)
50–59	246,858 (23.3)	2,622 (5.5)
60–69	248.681 (23.5)	7,060 (14.7)
70–79	202,083 (19.1)	16,591(34.7)
80+	128,636 (12.1)	20,504 (42.8)
**Year of diagnosis**		
1992–1997	140,189 (13.2)	15,279 (31.9)
1998–2003	269,769 (25.5)	18,320 (38.3)
2004–2008	277,068 (26.2)	10,027 (20.9)
2009–2014	372,022 (35.1)	4,246 (8.9)
**T stage**		
T0	1,014 (0.1)	16 (0.03)
T1	563,596 (53.2)	24,082 (50.3)
T2	264,690 (25.0)	11,432 (23.9)
T3	44,764 (4.2)	1,477 (3.1)
T4	30,755 (2.9)	1,706 (3.6)
Tis	1,625 (0.2)	109 (0.2)
Tmic	18,966 (1.8)	608 (1.3)
TX adjusted	81,940 (7.7)	7,166 (15.0)
Any T, mets	50,065 (4.7)	1,203 (2.5)
Other	1633(0.2)	73 (0.2)
**N stage**		
N0	646,142 (61.0)	29,782 (62.2)
N1	211,141 (19.9)	6,930 (14.5)
N2	61,547 (5.8)	2,193 (4.6)
N3	43,745 (4.1)	1,179 (2.5)
NX adjusted	94,840 (9.0)	7,715 (16.1)
Other	1,633 (0.2)	73 (0.2)
**M stage**		
M0	972,635 (91.8)	43,961 (91.8)
M1	50,065 (4.7)	1,203 (2.5)
MX	34,715 (3.3)	2,635 (5.5)
Other	1,633 (0.2)	73 (0.2)
**Race**		
White	865,759 (81.7)	41,180 (86.0)
African American	107,108 (10.1)	4,753 (9.9)
Other	80,347 (7.6)	1,789 (3.7)
Unknown	5,834 (0.6)	150 (0.3)
**Laterality**		
Left	514,685 (48.6)	23,016 (48.1)
Right	531,516 (50.2)	23,949 (50.0)
Other	12,847 (1.2)	907 (1.9)
**Receipt of radiotherapy**		
Beam radiation	449,318 (42.4)	13,259 (27.7)
None/unknown	547,376 (51.7)	32,492 (67.9)
Other	62,354 (5.9)	2,121 (4.4)
**Receipt of chemotherapy**		
Yes	391,296 (36.9)	5,657 (11.8)
No/unknown	667,752 (63.1)	42,215 (88.2)

*Database “SEER 18 Regs Research Data + Hurricane Katrina Impacted Louisiana Cases, Nov 2017 Sub (1973–2015 varying)” was used.

*^†^Database “Incidence – SEER 18 Regs Custom Data (with additional treatment fields), Nov 2016 Sub (1973–2014 varying) – Linked To County Attributes – Total.”*

**FIGURE 1:**
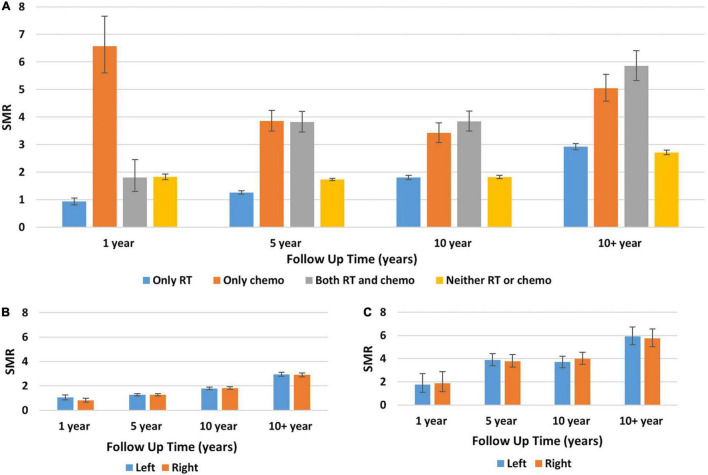
Standard mortality ratios for death from heart disease in breast cancer patients stratified by treatment received, disease laterality, and era diagnosed. **(A)** The *Y*-axis represents the SMR for death from heart disease and the *X*-axis depicts the time since diagnosis in years. Each color represents a different treatment received by patients: blue, only radiotherapy; orange, only chemotherapy; gray, both radiotherapy and chemotherapy; yellow, neither chemotherapy nor radiotherapy. Throughout the follow-up, the two patient groups with the highest SMR for death from heart disease were patients receiving only chemotherapy and patients receiving both radiotherapy and chemotherapy. **(B,C)** The *Y*-axis represents SMR for death from heart disease and the *X*-axis depicts the time since diagnosis in years. Each color represents patients with either left-sided primary disease (blue) or right-sided primary disease (orange). Panel **(B)** shows patients who received only radiotherapy and panel **(C)** is patients who received both radiotherapy and chemotherapy. For all timepoints and treatment groups, the SMR for death from heart disease was similar regardless of disease laterality. Generated from SEER Database: Incidence – SEER 13 Regs excluding AK Custom Data (with additional treatment fields), Nov 2016 Sub (1992–2014) for SMRs – Linked To County. MP-SIR session.

For the primary objective, the SMR for death from heart disease was evaluated for breast cancer patients based on the treatment received over time. Figure 1 shows SMR for death from heart disease based on treatment group and laterality of disease. Figure 1A shows that at one year post diagnosis, patients receiving only chemotherapy had the highest SMR for death from heart disease with a value of 6.57 (95% CI 5.60–7.66, *p* < 0.001). The SMR was lowest at this time point for patients who received only radiotherapy with a value of 0.93 (95% CI 0.81–1.06) however given the CI this was not statistically significant. At 10+ years, the highest SMR was in patients who received both chemotherapy and radiotherapy, with a value of 5.85 (95% CI 5.32–6.41, *p* < 0.001). The next highest SMR at this time point was in patients receiving only chemotherapy. Figures 1B,C demonstrate no statistically significant difference in the SMR for death from heart disease based on disease laterality. Compared to the general population, patients with left-sided disease treated with radiotherapy had an SMR for death from heart disease at 10+ years of 2.94 (95% CI 2.78–3.11, *p* < 0.001) and patients with right-sided disease treated with radiotherapy had an SMR of 2.90 (95% CI 2.74–3.07, *p* < 0.001) at 10+ years. A similar trend was also seen in patients who received both chemotherapy and radiotherapy.

Supplementary Figure 1 compares SMR pre 2000 (panel A) and post 2000 (panel B) for breast cancer patients who received radiotherapy to those who did not. It shows that at each follow-up time point the SMR for death from heart disease was comparable for patients regardless of if they received radiotherapy or not. Importantly, Supplementary Figures A,B cannot be compared to one another as they represent different United States reference populations and therefore should not be compared against each other.

**FIGURE 2:**
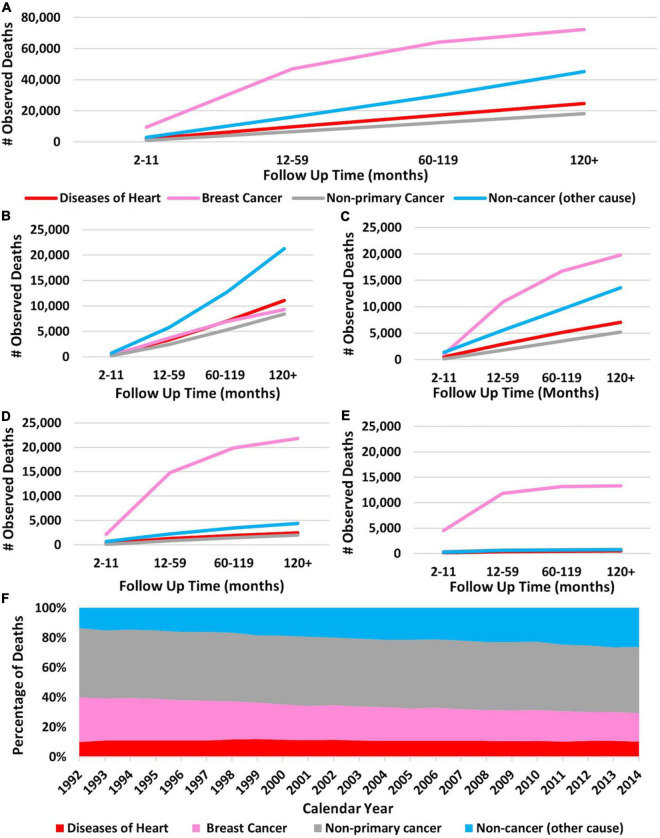
Cumulative death in breast cancer patients over time broken down by cause of death and stage of disease. The *Y*-axis of each panel shows the observed number of deaths and the *X*-axis shows the time since diagnosis in months. Each line represents a specific cause of death with the following colors: red, heart disease; pink, breast cancer; gray, non-primary cancer; and blue, non-cancer cause. **(A)** Cumulative number of deaths in all breast cancer patients (regardless of stage) separated by cause of death over time. Breast cancer was the leading cause of death at all time points. **(B)** Cause of death in stage I patients according to the Adjusted AJCC 6th Edition. **(C)** Cause of death in stage II patients. **(D)** Cause of death in stage III patients. **(E)** Cause of death in stage IV patients. Breast cancer was the leading cause of death in all patients, except those with stage 1 disease. Heart disease caused the most fatalities in stage I patients at 10+ years follow-up. Panel **(F)** shows the percentages of cause of death for each calendar year throughout the study period. The *X*-axis represents calendar years and the *Y*-axis represents percent of death per year. Pink represents breast cancer, gray is non-breast cancer, blue is non-cancer causes, and red is heart disease. Brest cancer accounts for the most deaths followed by non-cancer causes. As calendar year increases the percentage of patients dying from breast cancer decreases and the percentage of patients dying from non-cancer causes increases. Heart disease consistently accounts for at least 16% of cause of death. Panels **(A–E)**: generated from SEER Database: Incidence – SEER 13 Regs excluding AK Custom Data (with additional treatment fields), Nov 2016 Sub (1992–2014) for SMRs – Linked To County. MP-SIR session. Panel **(F):** generated from Incidence-Based Mortality – SEER 9 Regs Research Data, Nov 2018 Sub (1975–2016) <Katrina/Rita Population Adjustment> – Linked To County. Rate session type.

Figures 2A–E show the cumulative number of deaths throughout the study period, separated by cause of death. Importantly, in this figure, non-cancer causes and heart disease are separate categories. At 10+ years follow-up, 24,725 patients died from heart disease, 45,175 patients died from non-cancer causes, 72,459 patients died from breast cancer, and 18,172 patients died from non-primary cancer. Overall, breast cancer was the leading cause of death, followed by non-cancer causes and heart disease. According to Figure 2A, 28% of patients who died from non-breast cancer causes died from heart disease at 10+ years. Figure 2F shows the percentages of deaths for each calendar year separated by cause of death. Heart disease consistently accounts for at least 10% of cause of death and non-breast cancer causes of death consistently accounts for the largest percentage of deaths. Of note, newly diagnosed breast cancer patients are included in each year’s total, therefore the totals for death from heart disease become artificially low. This is represented in Supplementary Figure 2, which provides a schematic representation for the creation of Figure 2F.

For the secondary objective, ORs and HRs were calculated from multivariable models to evaluate the risk of death from heart disease compared to other breast cancer patients. Table 2 shows that the HRs for fatal heart disease increased as patient age increased, 35.72 (95% CI 33.72–38.12, *p* < 0.001) for patients older than 80 years compared to patients <49 years of age. Both receipt of chemotherapy and receipt of radiotherapy showed lower HRs compared to the reference group and laterality did not show a statistically significant difference with regards to left vs. right-sided disease and risk of fatal heart disease. The HR decreased as the year of diagnosis increased; patients diagnosed from 2009 to 2014 had a HR of 0.36 (95% CI 0.35–0.37, *p* < 0.001) compared to the reference group of patients diagnosed from 1992 to 1997. The risk of fatal heart disease was highest in patients with smaller tumors; HR 1.15 (95% CI 1.12–1.18) for tumors stage T2. OR showed the same trends as for the HRs mentioned.

**TABLE 2:** Odds ratios and hazard ratios of fatal heart disease among cancer patients.

	Multivariable logistic regression model			Multivariable fine and gray’s model		
	ORs	95% CI	*p*-value^‡^	HRs	95% CI	*p*-value^‡^
**Year of diagnosis**						
1992–1997	1.00	–		1.00	–	
1998–2003	0.60	0.58–0.61	<0.001	0.74	0.72–0.75	<0.001
2004–2008	0.30	0.29–0.31	<0.001	0.51	0.50–0.52	<0.001
2009–2014	0.08	0.08–0.09	<0.001	0.36	0.35–0.37	<0.001
**T stage**						
T1	1.00	–		1.00	–	
T2	1.14	1.11–1.17	0.252	1.15	1.12–1.18	<0.001
T3–T4	1.10	1.05–1.15	0.469	1.14	1.10–1.19	<0.001
TX adjusted	1.23	1.09–1.39	0.023	1.12	1.01–1.26	0.03
**N stage**						
N0	1.00	–		1.00	–	
N1	0.97	0.94–1.00	0.126	0.98	0.95–1.00	0.095
N2–N3	0.93	0.89–0.97	0.549	0.96	0.93–1.00	0.083
NX adjusted	0.87	0.77–0.97	0.07	1.00	0.89–1.12	0.987
**Age**						
<49 years	1.00	–		1.00	–	
50–59 years	2.43	2.26–2.62	<0.001	2.42	2.25–2.60	<0.001
60–69 years	6.63	6.20–7.10	0.013	6.54	6.12–6.99	<0.001
70–79 years	17.61	16.49–18.80	<0.001	16.77	15.73–17.89	<0.001
80+ years	38.56	36.09–41.21	<0.001	35.72	33.47–38.12	<0.001
**Race**						
White	1.00	–		1.00	–	
African American	1.41	1.36–1.46	<0.001	1.37	1.33–1.42	<0.001
Other/unknown	0.74	0.71–0.78	<0.001	0.77	0.73–0.81	<0.001
**Laterality**						
Right-origin of primary	1.00	–		1.00	–	
Left-origin of primary	1.00	0.98–1.02	0.946	1.00	0.98–1.02	0.828
**Receipt of chemotherapy**						
No/unknown	1.00	–		1.00	–	
Yes	0.71	0.69–0.74	<0.001	0.73	0.71–0.76	<0.001
**Receipt of radiotherapy**						
None/unknown	1.00	–		1.00	–	
Yes	0.75	0.74–0.77	<0.001	0.75	0.74–0.77	<0.001

*^‡^p-Value reported from Type III Wald test.*

**FIGURE 3:**
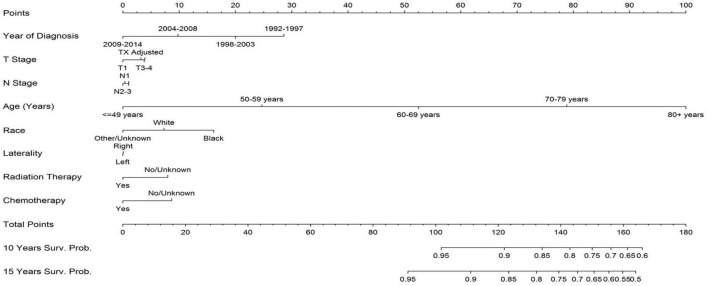
Nomogram for likelihood of breast cancer patients dying from heart disease. The two greatest predictors of death from heart disease are earlier year of diagnosis and older age. Disease laterality does not provide meaning predictive insight into death from heart disease. Receipt of chemotherapy and radiation therapy have a relative minimal impact of death from heart disease; in this part of the analysis they have a paradoxical protective effect because of bias in selecting patients for the treatment. To use the nomogram, sum each patient’s characteristic to get a total point sum which can then be used to predict the patient’s survival probability.

Figure 3 represents a nomogram for the study population and shows that patient age was the greatest predictor for cardiac mortality in breast cancer patients. Earlier year of diagnosis also predicted death from heart disease: patients diagnosed from 1992 to 1997 had the greatest likelihood of death from heart disease and patients diagnosed from 2009 to 2014 had the lowest likelihood of cardiac mortality. Tumor stage and disease laterality were weak predictors for likelihood of death from cardiac disease.

## Discussion

The goal of the current study is to determine the risk of fatal heart disease in breast cancer patients and to determine the best predictive factors for this risk. Our analysis shows that 47,872 patients (4.6%) diagnosed from 1992 to 2014 died of heart disease. The risk is greatest among older women (i.e., >70 years) with small tumors (i.e., <2 cm) and longer follow-up (i.e., >5–10 years) who receive chemotherapy. Modern radiotherapy has minimal impact on death from heart disease. This study is an important addition to the current body of literature as it helps highlight which patients are at greatest risk from cardiac related mortality secondary to cancer treatment. Our study is unique from other retrospective studies due to the large sample size and follow-up duration further strengthening our analysis.

Progress in breast cancer treatment has improved the long-term survival for breast cancer patients and as a result more patients die from other causes, commonly heart disease (9, 14–16). Darby et al. demonstrates that at 20+ follow-up years patients receiving radiotherapy have an 8.2% (95% CI 0.4–26.6) increase in the rate of major coronary events per Gray of mean heart dose (17). Henson et al. show that the risk of cardiac related mortality was highest for patients treated with radiation in earlier decades; specifically for patients who received radiotherapy from 1973 to 1982, the cardiac mortality ratio was 1.90 (1.52–2.37) 20+ years after diagnosis (16). Our study builds on these findings, by studying the risk of fatal heart disease in the short- and long-term. We show that the risk for fatal heart disease begins to increase at 5 years follow-up, and peaks at 10+ years. The highest SMR for all treatment groups is seen at 10+ years except for the chemotherapy only group which peaks at 1 year.

To address the primary objective, we show that chemotherapy is associated with a greater risk of fatal heart disease than radiotherapy. Patients who receive chemotherapy have higher SMRs for death from heart disease at all time points than patients who receive only radiotherapy. This is consistent with Bradshaw et al.’s discussion who show that patients treated with chemotherapy had higher HRs for cardiovascular mortality compared to patients treated with radiotherapy. They found that the HR for cardiovascular specific mortality was 1.7 (95% CI 1.1–2.6) while those receiving radiotherapy had an HR of 1.2 (95% CI 0.82–1.7) (18).

The risk of death from heart disease after radiotherapy is suspected to be higher in patients with left-sided disease as these patients typically receive on average higher radiation doses to the heart compared to patients with right-sided disease (10, 17, 19). Li et al., shows that there is no statistically significant difference between disease laterality and risk of cardiac-related mortality, as the HRs for right vs. left-sided tumor is 1.025 (95% CI 0.856–1.099, *p* = 0.484) (20). Our analysis similarly does not observe a statistically significant difference for cardiac mortality on the basis of disease laterality, shown in Figures 1B,C, 3.

Progress in radiotherapy techniques can help explain why the risk of fatal heart disease is lower in patients treated in the modern era. Newer techniques, such as partial breast irradiation, are more targeted, limiting exposure of the heart to radiation (21, 22). With regards to treatment era, Table 2 and Figure 3 show that year of diagnosis is a strong predictor for death from heart disease, with patients diagnosed in earlier years being at an increased risk of fatal heart disease. Modern radiotherapy techniques for breast cancer have consistently reduced incidental cardiac dose, which can help explain the observation reported herein (23). However it should be noted that many factors beyond radiotherapy technologies, such as improved drug development and cancer staging, have also changed since 2000 and as such it is difficult to ascertain the exact degree that newer radiotherapy techniques specifically have had on cardiac disease mortality. In addition, we cannot exclude the possibility of immortal time bias for earlier-cohort patients. As follow-up time increases, there will also be a higher incidence of cardiac mortality because the overall risk of heart disease increases with patient age. Therefore, patients with both an earlier year of diagnosis and longer follow-up are at an increased risk for death from heart disease. As the length of follow-up increases, the competing risk for death from non-breast cancer causes increases, of which heart disease is a major cause.

To address the secondary objective, we calculate HRs and ORs to evaluate the risk of fatal heart disease compared to other breast cancer patients. According to Table 2 the risk of fatal heart disease was associated with increased patient age, earlier year of diagnosis, and African American race. Disease laterality did not have a statistically significant difference on risk of heart disease. The counterintuitive results of Table 2 showing a seemingly protective role for chemotherapy and radiotherapy can be explained by selection bias. Patients with significant cardiac mortality risk are less likely to receive radiotherapy or chemotherapy. Conversely, healthier patients are more likely to receive radiotherapy or chemotherapy. As a result of this selection bias, there appears to be a paradoxical decreased risk of fatal heart disease with chemotherapy and radiotherapy. We also show that patients with smaller tumors are more likely to die from heart disease compared to those with larger tumors. This can be explained because as time progresses, patients with smaller tumors are more likely to die of a cause other than their breast cancer, and of those causes, heart disease is a common cause.

There are some limitations to our study as this is a retrospective study over the past 40 years and treatment has changed. Using the SEER database has some limitations particularly regarding treatment details. We do not have access to chemotherapy agents used, dosages, or timing of treatment. Similar limitations apply for radiotherapy dosing and timing. Also, treatment in SEER is coded as no/unknown and therefore it is possible that some of the patients coded as unknown received therapy.

Additionally, we do not have access to baseline cardiac function in these patients or information about risk factors for heart disease, such as obesity or smoking. Further information regarding other cardiac commodities would help strengthen the analysis but are unavailable in SEER.

Finally, there are limitations with how heart disease is coded in SEER. Heart disease as coded in SEER encompasses a variety of conditions that affect the heart ranging from hypertensive heart disease to conduction disorders and further specification is not available. Therefore, we do not have more specific information beyond heart disease for cause of death and we are unable to say if death was due to myocardial ischemia or heart failure or another cause of cardiac related mortality as they are all coded under heart disease.

## Conclusion

In conclusion, 5–10% of breast cancer patients die of heart disease. The greatest mortality rate we observe in our analysis are patients of older age (i.e., >70 years), smaller tumor size (i.e., <2 cm), longer duration of follow-up, and receipt of chemotherapy. Cardio-oncology clinics can better determine which patients need long term follow-up for prevention of death from cardiovascular disease.

## Data Availability Statement

Publicly available datasets were analyzed in this study. This data can be found here: https://seer.cancer.gov/data-software/linked_databases.html; SEER 18 Regs Research Data+Hurricane Katrina Impacted Louisiana Cases, Nov 2017 Sub (1973–2015 varying) database Incidence – SEER 18 Regs Custom Data (with additional treatment fields), Nov 2016 Sub (1973–2014 varying) – Linked To County Attributes – Total database.

## Author Contributions

NZ and AA were involved in study design, data collection, interpretation, and writing the manuscript. XW, MW, and EL were involved with biostatistics, data interpretation, and writing portions the manuscript. SH, JR, DT, RD, and AL were in involved in data interpretation and manuscript editing. All authors contributed to the article and approved the submitted version.

## Conflict of Interest

AL received honoraria from AstraZeneca for advisory board and speaker’s fees, unrelated to this work. The remaining authors declare that the research was conducted in the absence of any commercial or financial relationships that could be construed as a potential conflict of interest.

## Publisher’s Note

All claims expressed in this article are solely those of the authors and do not necessarily represent those of their affiliated organizations, or those of the publisher, the editors and the reviewers. Any product that may be evaluated in this article, or claim that may be made by its manufacturer, is not guaranteed or endorsed by the publisher.

## Footnotes

1
https://seer.cancer.gov/seerstat/

## Supplementary Material

Supplementary Figure 1 **(A,B)** The *Y*-axis represents SMR for death from heart disease and the *X*-axis depicts the time since diagnosis in years. Each color represents patients either treated with no radiation (blue) or treated with radiotherapy (orange). Panel **(A)** are patients diagnosed before 2000 and panel **(B)** are patients treated post 2000. Patients were selected only based on their RT status and chemotherapy status was not accounted for in this figure. In both eras, the patients treated with radiotherapy and those not treated with radiotherapy have similar SMR for death from heart disease. Of note, the two panels should not be compared to one another as SMRs from different time periods cannot be compared to one another as they represent different time periods and therefore the reference population may be different from each time period. Generated from SEER Database: Incidence – SEER 13 Regs excluding AK Custom Data (with additional treatment fields), Nov 2016 Sub (1992–2014) for SMRs – Linked To County. MP-SIR session.

Supplementary Figure 2 Schematic representation for how Figure 2F was generated.
