# Supplementary figures and images for: Long-Term Risk of Death From Heart Disease Among Breast Cancer Patients
Source: Front Cardiovasc Med. 2022 Apr 13;9:784409. doi: 10.3389/fcvm.2022.784409 (PMC9043135; doi:10.3389/fcvm.2022.784409)

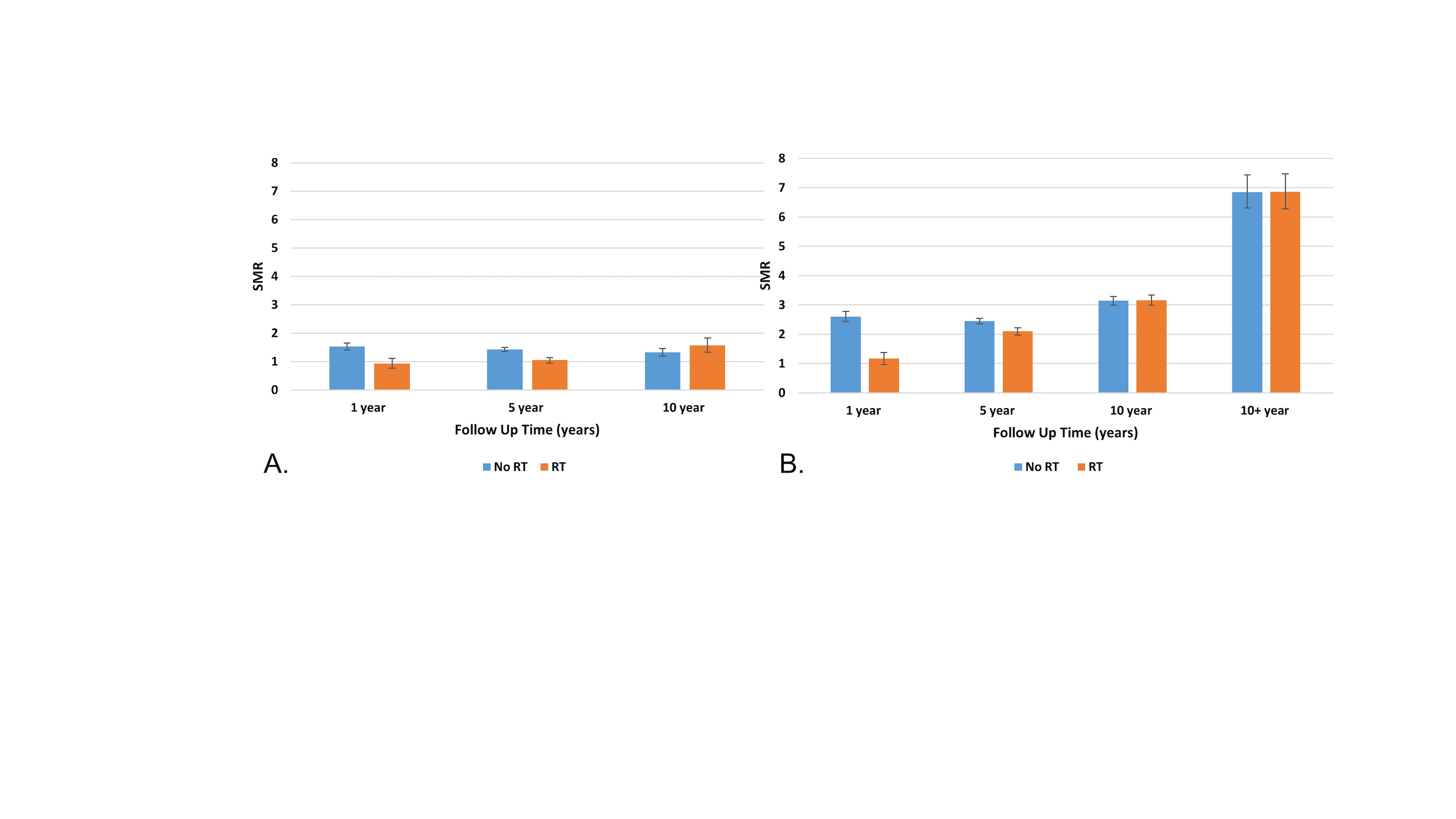

Supplement: Supplementary Figure 1 — (A,B) The Y-axis represents SMR for death from heart disease and the X-axis depicts the time since diagnosis in years. Each color represents patients either treated with no radiation (blue) or treated with radiotherapy (orange). Panel (A) are patients diagnosed before 2000 and panel (B) are patients treated post 2000. Patients were selected only based on their RT status and chemotherapy status was not accounted for in this figure. In both eras, the patients treated with radiotherapy and those not treated with radiotherapy have similar SMR for death from heart disease. Of note, the two panels should not be compared to one another as SMRs from different time periods cannot be compared to one another as they represent different time periods and therefore the reference population may be different from each time period. Generated from SEER Database: Incidence – SEER 13 Regs excluding AK Custom Data (with additional treatment fields), Nov 2016 Sub (1992–2014) for SMRs – Linked To County. MP-SIR session. [file Image_1.tiff]

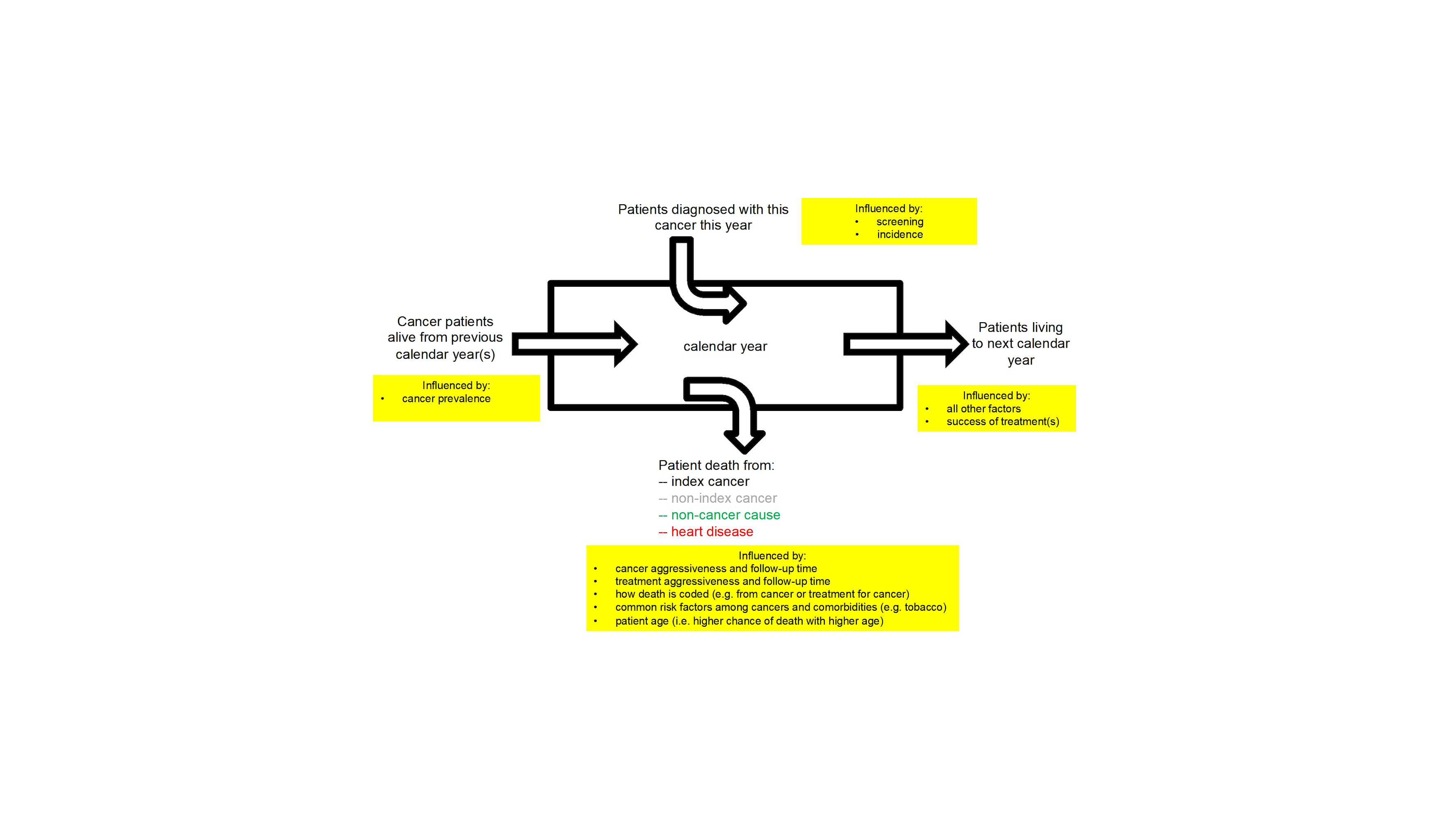

Supplement: Supplementary Figure 2 — Schematic representation for how Figure 2F was generated. [file Image_2.tiff]
