# Supplementary material for: Long-Term Risk of Death From Heart Disease Among Breast Cancer Patients
Source: Front Cardiovasc Med. 2022 Apr 13;9:784409. doi: 10.3389/fcvm.2022.784409 (PMC9043135; doi:10.3389/fcvm.2022.784409)
Supplement: Supplementary file 3 [file Data_Sheet_1.docx]

Data for table 1 was abstracted using a case listing session.

Filename:

SEER*Stat Version:

8.3.6

Date: 27-Oct-19

Session Type:  Case Listing

SUGGESTED CITATION

Software:  Surveillance Research Program, National Cancer Institute SEER*Stat software (www.seer.cancer.gov/seerstat) version 8.3.6.

Data:  Surveillance, Epidemiology, and End Results (SEER) Program (www.seer.cancer.gov) SEER*Stat Database: Incidence - SEER 18 Regs Custom Data (with additional treatment fields), Nov 2016 Sub (1973-2014 varying) - Linked To County Attributes - Total U.S., 1969-2015 Counties, National Cancer Institute, DCCPS, Surveillance Research Program, released April 2017, based on the November 2016 submission.

DATA

Database:  Incidence - SEER 18 Regs Custom Data (with additional treatment fields), Nov 2016 Sub (1973-2014 varying) - Linked To County Attributes - Total U.S., 1969-2015 Counties

SELECTION

Select Only:  Malignant Behavior, Known Age, Cases in Research Database

Case:   {Race, Sex, Year Dx, Registry, County.Year of diagnosis} = '1992',  '1993',  '1994',  '1995',  '1996',  '1997',  '1998',  '1999',  '2000',  '2001',  '2002', '2003',  '2004',  '2005',  '2006',  '2007',  '2008',  '2009',  '2010',  '2011',  '2012',  '2013',  '2014'

AND {Site and Morphology. Site recode ICD-O-3/WHO 2008} = 'Breast'

TABLE

Column:  Breast - Adjusted AJCC 6th T (1988+)

Breast - Adjusted AJCC 6th N (1988+)

Breast - Adjusted AJCC 6th M (1988+)

Laterality

Age recode with <1 year olds

Race recode (White, Black, Other)

Year of diagnosis

Chemotherapy recode (yes, no/unk)

Radiation recode

Vital status recode (study cutoff used)

COD to site recode

Data for column 2 of table 1 was abstracted using a case listing session with cause of death as heart disease.

Filename:

Case Listing Session-1 Matrix-1

SEER*Stat Version:

8.3.6

Date: 19-Feb-20

Session Type: Case Listing

SUGGESTED CITATION

Software: Surveillance Research Program, National Cancer Institute SEER*Stat software (www.seer.cancer.gov/seerstat) version 8.3.6.

Data: Surveillance, Epidemiology, and End Results (SEER) Program (www.seer.cancer.gov) SEER*Stat Database: Incidence - SEER 18 Regs Custom Data (with additional treatment fields), Nov 2016 Sub (1973-2014 varying) - Linked To County Attributes - Total U.S., 1969-2015 Counties, National Cancer Institute, DCCPS, Surveillance Research Program, released April 2017, based on the November 2016 submission.

DATA

Database: Incidence - SEER 18 Regs Custom Data (with additional treatment fields), Nov 2016 Sub (1973-2014 varying) - Linked To County Attributes - Total U.S., 1969-2015 Counties

SELECTION

Select Only: Malignant Behavior, Known Age, Cases in Research Database

Case: {Site and Morphology.Site recode ICD-O-3/WHO 2008} = 'Breast'

AND {Race, Sex, Year Dx, Registry, County.Year of diagnosis} = '1992', '1993', '1994', '1995', '1996', '1997', '1998', '1999', '2000', '2001', '2002', ‘2003', '2004', '2005', '2006', '2007', '2008', '2009', '2010', '2011', '2012', '2013', '2014'

AND {Cause of Death (COD) and Follow-up.COD to site rec KM} = 'Diseases of Heart'

TABLE

Column:

Breast - Adjusted AJCC 6th Stage (1988+)

Breast - Adjusted AJCC 6th T (1988+)

Breast - Adjusted AJCC 6th N (1988+)

Breast - Adjusted AJCC 6th M (1988+)

Age recode with <1 year olds

Chemotherapy recode (yes, no/unk)

Radiation recode

Race recode (White, Black, Other)

Laterality

Year of diagnosis

Data for figure 1 was abstracted using MP-SIR session to obtain SMRs. This same session was run multiple times changing only the chemotherapy and radiation therapy coding in order to obtain all 4 possibilities of treatment groups (only chemotherapy, only RT, both chemotherapy and RT or neither chemotherapy nor RT).

Filename:

MP-SIR Session-2 Matrix-3

SEER*Stat Version:

8.3.6

Date:17-Oct-19

Session Type: MP-SIR

SUGGESTED CITATION

Software: Surveillance Research Program, National Cancer Institute SEER*Stat software (www.seer.cancer.gov/seerstat) version 8.3.6.

Data: Surveillance, Epidemiology, and End Results (SEER) Program (www.seer.cancer.gov) SEER*Stat Database: Incidence - SEER 13 Regs excluding AK Custom Data (with additional treatment fields), Nov 2016 Sub (1992-2014) for SMRs - Linked To County Attributes - Total U.S., 1969-2015 Counties, National Cancer Institute, DCCPS, Surveillance Research Program, released April 2017, based on the November 2016 submission.

DATA

Database: Incidence - SEER 13 Regs excluding AK Custom Data (with additional treatment fields), Nov 2016 Sub (1992-2014) for SMRs - Linked To County Attributes - Total U.S., 1969-2015 Counties

RATES

Name: U.S. Mortality 1973-2014 (Nov 2016 sub), Race (WU/B/O), Event: COD rec (HIV grouped w/oth infectious)

See rate session page(s) below.

SELECTION

Index Record: First Primary Only (Sequence Number 0 or 1)

Select Only: Malignant Cases, Known Age

Exclude All Death Certificate and Autopsy Only

{Race, Sex, Year Dx, Registry, County.Year of diagnosis} = '1992', '1993', '1994', '1995', '1996', '1997', '1998', '1999', '2000', '2001', '2002', '2003','2004', '2005', '2006', '2007', '2008', '2009', '2010', '2011', '2012', '2013', '2014'

AND {Site and Morphology.Site recode ICD-O-3/WHO 2008} = 'Breast'

AND {Therapy.Chemotherapy recode (yes, no/unk)} = 'No/Unknown', 'Yes'

AND {Therapy.Radiation recode} = 'None/Unknown'

PARAMETERS

Exposure Date: Date of diagnosis recode

Latency Exclusion: 2 months

Start Date: Date of diagnosis recode

Cutoff Start: Jan 1992

Cutoff End: Dec 2014

Latency: 1y, 5y, 10y

EVENTS

Analysis Type: Single Outcome Analysis

Exit Point: Exit at Any Event in Rate File

Event possible on Index Record

Selected Events: COD rec (HIV grouped w/oth infectious) (Event Variable)

\

STATISTICS

Table Type: SIR Tables

Include: 95% Confidence Intervals (Exact Method)

TABLE

Page: Chemotherapy recode (yes, no/unk)

Row: COD to site recode

Column: Latency [*calculated*]

USER DEFINITIONS

COD rec (HIV grouped w/oth infectious) (Event Variable) [Cause of death recode]

OUTPUT

Precision: 2 decimal places (default)

Historic: Not Selected

Rates: Rate Session
